# Guiding deployment of resistance in cereals using evolutionary principles

**DOI:** 10.1111/eva.12175

**Published:** 2014-06-11

**Authors:** Jeremy J Burdon, Luke G Barrett, Greg Rebetzke, Peter H Thrall

**Affiliations:** 1 CSIRO, Plant IndustryCanberra, ACT, Australia; 2 CSIRO Biosecurity FlagshipCanberra, ACT, Australia

**Keywords:** adult plant resistance, aggressiveness, gene deployment, gene-for-gene, infectivity, minor gene resistance, mixtures, resistance, spatial deployment.

## Abstract

Genetically controlled resistance provides plant breeders with an efficient means of controlling plant disease, but this approach has been constrained by practical difficulties associated with combining many resistance genes together and strong evolutionary responses from pathogen populations leading to subsequent resistance breakdown. However, continuing advances in molecular marker technologies are revolutionizing the ability to rapidly and reliably manipulate resistances of all types – major gene, adult plant and quantitative resistance loci singly or multiply into individual host lines. Here, we argue that these advances provide major opportunities to deliberately design deployment strategies in cereals that can take advantage of the evolutionary pressures faced by target pathogens. Different combinations of genes deployed either within single host individuals or between different individuals within or among crops, can be used to reduce the size of pathogen populations and generate patterns of disruptive selection. This will simultaneously limit immediate epidemic development and reduce the probability of subsequent evolutionary change in the pathogen for broader infectivity or increased aggressiveness. The same general principles are relevant to the control of noncereal diseases, but the most efficacious controls will vary reflecting the range of genetic options available and their fit with specific ecology and life-history combinations.

## Introduction

Growth in modern agricultural productivity has been underpinned to a large extent by the utilization of effective strategies to control disease. However, by virtue of their high plant densities, low diversity, large areas and high nutrient and water availability, agricultural crops are particularly prone to damaging attack by a range of fungal pathogens. To counter potential losses, disease resistance breeding has been a major component of crop improvement programmes ever since Biffen first demonstrated single gene inheritance of resistance to *Puccinia striiformis* in wheat (Biffen 1905). Due to its immediate and qualitative efficacy, much of the initial research and breeding focus was centred on the use of major gene resistance to protect crops from attack by specific races of pathogens although it became increasingly recognized that other race-nonspecific resistance existed and was subsequently included in breeding strategies. Despite this, evolution in the target pathogen for changed infectivity has repeatedly led to the failure of resistance in many crop species (Johnson 1961).

Disease resistance has been categorized using a plethora of terms [e.g. horizontal, race nonspecific, multigenic, quantitative, partial, minor gene; vertical, race specific, major gene], but in essence, the variation that is generally available and used by breeders can be grouped into that which is race-specific (major gene) and that which is nonspecific being expressed against all races of a pathogen. Race-specific, major gene resistance generally complies with the gene-for-gene paradigm (Flor 1956) which is based on control by single genes; nonspecific resistance, on the other hand, is frequently thought of as multigenic although this is not always the case (Parlevliet 1993). Indeed, an increasingly important source of resistance in wheat, adult plant resistance (APR), is controlled by the action of single genes (see Box 1).

**Box 1: Resistance, infectivity and aggressiveness**

Pathogen infectivity and aggressiveness profiles are essentially the result of co-evolutionary interactions between pathogens and their host plants. Infectivity determines whether or not a pathogen will infect a given host and generate a fully compatible (susceptible) reaction. Pathogen isolates that generate a full susceptible reaction are considered infective while those that do not, (either failing to generate macroscopic symptoms or only limited infection and sporulation) are classed as noninfective. Host plants in these reactions are classed as susceptible, (fully) resistant or partially resistant respectively.

The genetic basis behind these phenotypic patterns is variable. Interactions that involve pathogen race-specific interactions with host lines typically involve gene-for-gene interactions with resistance control by single (major R) genes. Partial resistance on the other hand may be generated by any of (i) incompletely expressed R genes, (ii) adult plant resistance (APR) genes, or (iii) minor resistance genes. In the case of incompletely expressed major R genes, the interaction between host and pathogen is still race-specific; whereas for APR genes and resistance genes it is race nonspecific with the resistance being expressed against all pathogen isolates.

On the pathogen side, once infection occurs, aggressiveness is a quantitative component of measure of pathogen development on the host. While interactions may be race nonspecific, differences between pathogen isolates in a range of quantitative traits affecting epidemiologically relevant components of the pathogen's life-cycle (e.g. infection efficiency, latent period, infectious period, spore production etc.) may, when integrated over time, generate different levels of disease.

Partial resistance imposes different selective pressures on the pathogen than does R gene resistance. Furthermore, because it does not display the spectacular failures seen with R gene deployment, partial resistance is often regarded as ‘durable’ and therefore preferable. However, studies of such interactions have demonstrated than partial resistance does not prevent pathogen evolution rather, there is growing evidence for increased aggressiveness and adaptation by pathogen isolates to partially resistance host lines. The extent to which this occurs in important crop – pathogen interactions (e.g. rusts in wheat; blackleg in canola) – is unclear as is the fully integrated consequences of differences in different traits (e.g. latent period versus spore production per pustule) on epidemic development.

Major gene (R) resistance has largely been manipulated in breeding programmes through tracking of its phenotypic expression when challenged by individual pathogen races, bulk race inocula or exposure to natural field infection. However, the generally dominant nature of R gene expression imposes a major limitation on this approach as the phenotypic resistance expression of one gene may mask the resistance expression of another. This makes phenotypic tracking of two or more R genes only possible through the use of pathogen isolates capable of discriminating between the individual infection types of each R gene (McIntosh et al. 1995). Furthermore, in many cases, the expression of individual R genes may show strong genotype × environment interaction (G × E) effects with both host background and environment (especially temperature) affecting expression and resulting phenotype (McIntosh et al. 1995). Together, these issues, plus the need to assess some genes in the field (they are expressed in the adult plant rather than seedlings), impose significant time and cost constraints on the production of new varieties in which disease resistance is only one of potentially many traits being pursued (Pink 2002; Bonnett et al. 2005).

Response to the ‘boom-and-bust’ cycles (Johnson 1961) associated with novel R gene deployment followed by evolution of matching infectivity in the pathogen has seen increasing efforts invested in the use of race nonspecific minor gene resistance as a means of giving long-term durability to disease resistance. Indeed, increasingly, calls are being made for ‘the informed deployment of major, race-specific and partial, race-nonspecific resistance’ (Boyd et al. 2013) with strong arguments being mounted that ‘durability’ of resistance can only be achieved through the use of quantitative partial resistance with or without the presence of major R genes to reduce epidemic development. To date, practical experience generally supports these suppositions (Brun et al. 2010; Quenouille et al. 2013) – compare for example, the ongoing durability of minor gene resistance to black-leg of canola in Europe and Australia with the rapid (within 3–5 years) loss of protection from a range of major genes (Delourme et al. 2006; Sprague et al. 2006a).

Importantly, molecular technologies are providing (i) highly effective marker tagging systems for R genes and (ii) increasingly efficient means of manipulating multiple minor genes. As a consequence, in principle, few restraints are placed on the combinations of R genes, APR genes and minor gene resistances that are possible. However, what is lacking are careful assessments (both empirical and theoretical), using ecological and evolutionary principles, of the most effective disease resistance deployment strategies (including spatial considerations) that will maximize both the short-term epidemiological and the longer-term evolutionary benefits of different combination strategies. McDonald and Linde (2002a,b2002b) addressed one aspect of this question by considering some life-history attributes which increased the evolutionary potential of pathogens to overcome genetic resistance. Lannou (2012) also recognized this issue and underlined the complexities involved in understanding quantitative traits affecting pathogen aggressiveness and their epidemiological and evolutionary consequences. Here, we outline the range of gene deployment strategies available, their temporal and spatial options and the ways in which different combinations have radically different implications for pathogen evolution. While the epidemiological and evolutionary outcomes of some deployment strategies are already apparent, others have yet to be determined.

The current review particularly focuses on the use and deployment of different resistance mechanisms to control fungal pathogens of cereals. However, genetically based resistance mechanisms are widely used to counter the effects of a broad range of plant pathogens in agricultural, horticultural and forestry settings including field (e.g. cereals, legumes, oilseeds, potatoes) and vegetable crops (e.g. lettuce, brassicas, beans, tomatoes), pasture and amenity species (e.g. ryegrass, white clover, roses), perennial horticultural fruit crops (e.g. strawberries, raspberries, apples, grapevines), and a range of trees grown for timber or pulp (e.g. pines, poplars, eucalypts). As a consequence, while this review focuses on the control of cereal diseases, the application of an evolutionary approach to long-term disease control is equally applicable to most, if not all, agricultural disease control. Despite this, the nature of the specific controls invoked is likely to vary depending on the ecology and life history of both host and pathogen. For example, the extent of auto- versus allo-infection will alter between different hosts depending on their structural architecture. In turn, this will affect the balance of selection for infectivity versus aggressiveness. Furthermore, APR genes are an important part of the defence armoury of wheat and barley but have not been identified outside the Poaceae.

### Characteristics of major resistance mechanisms

**Figure 1:**
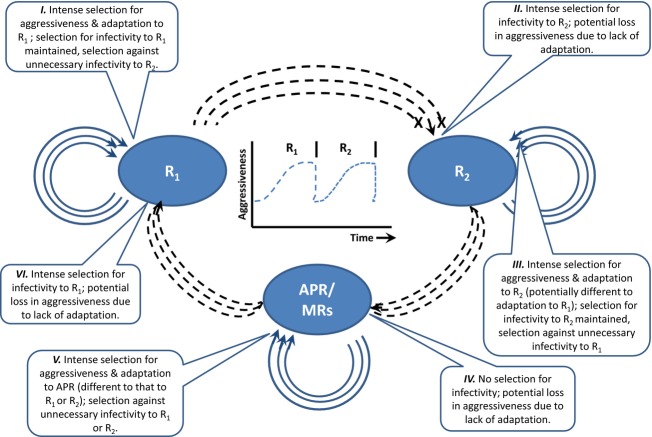
Changing patterns of selection imposed on pathogen populations by major gene resistance (R_1_, R_2_), adult plant resistance (APR) and partial resistance conferred by minor genes (MRs). As the cycle of interaction moves round from that with R_1_ to that with R_2_ and APR/MRs, pathogen isolates undergo fluctuations in the direction and intensity of selection for infectivity and aggressiveness [solid lines = auto-infection events; dashed lines = allo-infection events). The central graph provides a schematic of how pathogen aggressiveness might go through sequential increases and declines as R genes are overcome and selection for aggressiveness is re-enforced through auto-infection processes.

Disease symptoms, manifest as differences in lesion size and type, are the phenotypic expression of genetic interactions between host resistance and pathogen infectivity and aggressiveness modified by environmental conditions, including the host germplasm (‘genetic background’) in which resistance genes are expressed. Major R genes, mostly characterized by nucleotide-binding site leucine-rich repeat motifs (Dangl and Jones 2001; Meyers et al. 2005), are found in many plant species where they are effective against pathogen isolates lacking the matching infectivity allele. The resistance conferred by major R genes is typically characterized using nonlinear infection type (IT) scales with only the final few categories [i.e. on a 0–4 (cereal rusts) or 0–9 (mildews) scale] reflecting significant spore production (Johnson and Taylor 1976). Thus, even though major genes for resistance to rusts in cereals may permit some sporulation (i.e. race-specific partial resistance), such is the restraint on pathogen reproduction and transmission that it is only in favourable circumstances that this is likely to be sufficient to result in a disease epidemic. The same pattern of low–to-no pathogen reproduction associated with R genes is common to many other host–pathogen interactions (e.g. wild *Glycine* species challenged by *Phakopsora pachyrhizi*; Burdon and Speer 1984). This substantial reduction but not suppression of sporulation in incompatible reactions has significant evolutionary implications for the pathogen and is associated with intense selection for matching infectivity and a potential loss of aggressiveness due to lack of adaptation resulting from the presence of different minor genes and change in the genetic background in which they are expressed (see I.→II.: Fig. 1).

Although evidence for the existence of APR genes outside cereals is limited, APR is included as a separate category here because of its considerable significance in the protection of much of the world's major grain crops. Like major R gene resistance, APR is under simple single gene control, but unlike R genes confers a partial resistance that is effective against all pathogen isolates (i.e. is nonspecific). It should be noted that this resistance is very different to the partial resistance generated by incompletely expressed R genes; see Box 1. APR genes do not prevent sporulation, rather they slow the rate at which disease develops within the crop. From a breeding point-of-view, a particularly attractive feature of some APR genes in wheat is their effectiveness against several pathogens (e.g. *Lr*34/*Yr*18/*Pm*38, Lagudah et al. 2009; *Sr*2/*Lr*27/*Pm*?, Mago et al. 2011b; *Lr*67/*Yr*46/*Sr*55/*Pm*46), although others are effective against a single pathogen only (e.g. *Yr36* against stripe rust; Lagudah 2011).

Finally, quantitative resistance tends to be controlled by the action of multiple minor genes (two to 10 or more; Young 1996) each of which explain a fraction of the heritable variation (e.g. four quantitative trait loci (QTLs) controlling resistance to *Puccinia coronata* in *Lolium perenne* explain between 2.6% and 24.9% of the phenotypic variation (Muylle et al. 2005)). Like APR, this resistance is pathogen isolate nonspecific and acts to reduce the reproductive rate of the pathogen rather than prevent sporulation (Parlevliet 1978). Major R gene, APR and multiple minor genes may affect the size and evolutionary potential of pathogen populations in distinctly different ways. In situations where resistance completely prevents pathogen reproduction, pathogen survival will be restricted to those lineages whose infectivity matches the resistances present in the host population. Selection under this ‘viability, hard selection’ scenario will, depending on any associated fitness costs, favour preferential increase of novel pathotypes capable of overcoming resistances currently in the field – the potential ‘super-race’ phenomenon (Leonard 1977; Leach et al. 2001). On the other hand, APR and minor gene-controlled resistance, by permitting the survival of all races, are likely to reduce selection for the appearance of pathogens with greater infectivity (‘fecundity, soft selection’; IV. Fig. 1) although the direction and intensity of selection for aggressiveness will depend on any associated fitness costs (V. Fig. 1).

An interesting question arises as to whether selection on pathogens colonizing hosts incompletely expressing major R genes (partially resistant) will favour different pathogen traits depending on the degree to which pathogen sporulation is restricted. In situations where sporulation is almost completely restricted [e.g. pathogen isolate–host combinations resulting in low ITs (0–2)], there may be strong selection for novel infectivity rather than increased aggressiveness. In contrast, partial resistance that permits higher levels of sporulation (e.g. ITs = 2+ to 3) is epidemiologically more similar to minor gene-controlled resistance and hence is more likely to promote selection for increased pathogen aggressiveness. Complexities of this type are often encountered in host–pathogen interactions occurring in natural ecosystems (Antonovics et al. 2011).

## Costs of breeding for resistance

In developing new cultivars, breeders are confronted by a seemingly simple target – increasing yield as rapidly as possible while simultaneously ensuring varieties meet or exceeding quality standards. However, yield is highly variable depending on the biotic and abiotic environment. Hence, the simple goal of increasing yield rapidly becomes complex in light of diverse traits incorporated to fit the variety to the growing environment. For example, ability to cope with hostile soils (salinity, sodicity), flowering time requirements, increasing water-use efficiency, and pests and diseases are traits important in enhancing yield that may be positively (Burrows 1970) or negatively (Joshi and Chand 2002) associated with disease development.

In addition to these complexities, the expression of major resistance genes *per se* may also be associated with physiological costs that negatively affect agronomic performance. The extent of these ranges widely with a striking contrast between high costs of resistance often found in model systems and low costs in most crop systems (see the comprehensive review of Brown and Rant 2013). However, these authors also highlighted a number of studies showing that even in crop systems costs could be sufficiently large as to concern breeders (Summers and Brown 2013). Thus, the widely favoured *ml-o* gene resistance in barley has impacts on leaf length, necrotic flecking, grain size and number, and yield with yield reductions ranging from undetectable (Kølster and Stølen 1987) to low values of 4–5% (Kjær et al. 1990) to significant costs of 13–27% (Schwarzbach 1976). While values at the lower end of this range are out-weighed by the resistance provided against mildew epidemics, costs at the upper end limit the utility of such genes in normal agricultural production.

Less is known about costs associated with resistance genes effective against the three rust pathogens of wheat. However, a study of the effects of eight sources of stem rust resistance found that they were associated with reduced yield (The et al. 1988). In five cases, the differences between near-isogenic lines (BC5) with or without the resistance gene were nonsignificant, but in others, yield reductions of 3% (*Sr 24*) to 9% (*Sr26*) were detected. Both of these genes had been derived from wild crosses involving less or more distant relatives (*Thinopyrum ponticum* and *Agropyron elongatum* respectively). A similar association of reduced yield has been detected with resistance genes for eyespot and powdery mildew derived from *Aegilops speltoides* and *A. ventricosa* (Summers and Brown 2013). Given their origin, in both these studies, separating any costs associated with the genes themselves from yield depression associated with linked alien genetic material (i.e. linkage drag) is not easy.

It is important to recognize that costs associated with resistance are very often context dependent being influenced by the particular line in which they are expressed and the environment in which they are deployed (The et al. 1988; Brown 2002). As a consequence, relatively small costs do not rule out the use of particular genes in agriculture. Frequently, breeders are consciously or unconsciously able to compensate for these costs by selecting the best performing lines from those being assessed. For example, *Sr26* was used in the variety Kite which was one of the highest yielding varieties used in New South Wales, Australia in the 1970s, while the cultivar Sunelg – a Kite derivative contains both *Sr26* and *Sr24*!

### Epidemiological and evolutionary impacts on pathogens

Many factors are important in shaping the epidemiological and evolutionary behaviour of pathogen populations in space and time. Characteristics such as high mutation rates, mixed reproductive systems, high gene flow and large effective population sizes (McDonald and Linde 2002a; Barrett et al. 2008) are undoubtedly important drivers of the evolutionary potential of specific pathogens. However, basic attributes of pathogen dispersal are fundamentally important in affecting the frequency of encounter between specific infectivity factors and specific resistances (Thrall and Burdon 1997). Indeed, the cumulative sum of dispersal patterns of propagules from individual pustules within a crop and their fate – re-infection of the same host individual, of other hosts in the same or other crops, or of being lost entirely – is a key factor in determining the nature and intensity of evolutionary pressures being exerted by plants on their pathogens.

**Figure 2:**
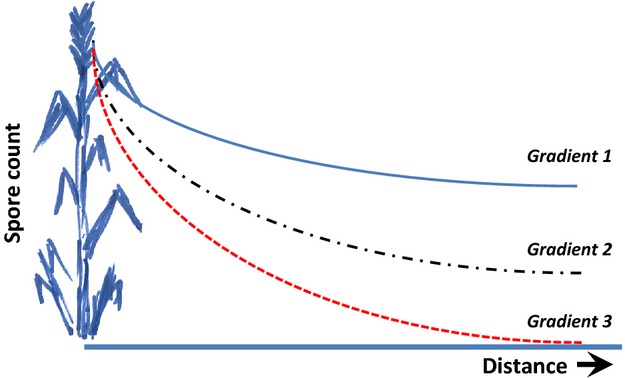
Dispersal patterns are a key feature in driving genetic interactions between pathogen and host. As the steepness of the dispersal gradient for propagules increases (G1 to G3), the extent of auto- versus allo-infection increases and the probability of dispersal from one host stand to another decreases.

Furthermore, while different pathogens may show distinctly different patterns of spatial distribution (often driven by their primary dispersal mode – wind, water, vector, etc.), realized dispersal patterns are highly environment dependent. For example, wind speed and gustiness will interact with features such as crop phenology and architecture (e.g. leaf area and degree of tillering) and the spatial position of the pustule in the crop to affect the frequency of allo- versus auto-infection (Burdon 1978; Fig. 2). Given that auto-infection means that the pathogen isolate in question already has the ability to infect the host, then auto-infection has the potential to maintain selective pressure on the pathogen for increasing aggressiveness. Potential for the evolution of increased aggressiveness on particular crop varieties has been shown through serial passaging of *Rhynchosporium secalis* and *Septoria nodorum* in barley (Abang et al. 2006) and wheat (Cunfer 1994), respectively. Variations in spore production (one measure of aggressiveness) have also been noted in *Puccinia striiformis* where, interestingly, an isolate of pathotype 41 E136 collected from the wheat variety Joss Cambier showed a higher level of spore production on that variety than did another isolate of the same pathotype collected on variety Cama (Johnson and Bowyer 1974). As plant pathogens rarely if ever kill their hosts so quickly as to prevent transmission, selection for reduced aggressiveness as is seen in some animal-viral systems (e.g. myxoma in rabbits; Fenner and Woodroofe 1965) is not likely. In contrast, allo-infection events involve infection of a new host individual that may or may not show the same resistance as the previous host. Where the original and the second hosts have the same resistance profiles (as typically occurs in modern pure-bred cultivars), these allo-infection events are, in a selective sense, the same as auto-infections and selection for aggressiveness will be maintained. On the other hand, where the second plant carries a different resistance specificity, selection on the pathogen will switch immediately to mortality selection with only individuals with the appropriate infectivity genes surviving.

In addition to the obvious advantage of being able to colonize a new host line what, if any, impact do mutations resulting in infectivity against R genes have on the pathogen? Host–pathogen coevolutionary models of gene-for-gene interactions have demonstrated the importance of trade-offs between infectivity and reproductive fitness for maintaining diversity in pathogen populations and preventing the emergence of a ‘super-race’ (Person 1966; Groth 1976; Leonard and Czochor 1980). Bearing in mind their potential to impose selective pressures of a different dimension on the evolutionary trajectory of an interaction (see III., Fig. 1), what evidence is there for such costs? If reproductive costs exist and are additive in effect, then comparisons of pathogen isolates carrying differing numbers of infectivity alleles should show an inverse correlation between fitness and infectivity measures. Such a relationship was found in a study of spore production per pustule generated by a wide range of isolates of *Melampsora lini* occurring in a native association with its host *Linum marginale* (Thrall and Burdon 2003). More recently, a detailed comparison of measures of latent period, pustule size and spore production per pustule in the interaction between *Puccinia coronata* and cultivated oats founds strong evidence for heritable genetic variation in these characters and trade-offs with pathogen infectivity levels (Bruns et al. 2014). However, the significance of measured aggressiveness values for population disease dynamics can be difficult to evaluate (Pariaud et al. 2012), and reduced fitness does not always segregate with infectivity (Bronson and Ellingboe 1985). Furthermore, there is a lack of knowledge regarding the outcomes of on-going auto-infection (sequential passaging) – over time does further interplay between host and pathogen result in increased fitness on that host line (spore production, infection efficiency etc.)? Comparison of relative spore efficacy among isolates of *Erysiphe graminis* f.sp. *tritici* suggests this might be the case (Villareal and Lannou 2000).

Finally, there is a major dichotomy between biotrophic and necrotrophic pathogens which also affects epidemiological outcomes. Necrotrophic pathogens interact with their hosts in a distinctly different way to biotrophic ones; their host range is frequently greater, and they are often able to survive in a saprophytic phase. Both these features can significantly alter population sizes and their dynamics through time, as well as the extent of potential evolutionary conflicts (cf. selection for survival on host versus survival as a saprophyte on dead tissue; Abang et al. 2006; Barrett et al. 2011; Sommerhalder et al. 2011). Thus, life-history attributes of pathogens are also an important consideration in determining epidemiological and evolutionary outcomes (Barrett et al. 2008).

## Deployment strategies – within field

### Single gene resistances

‘Boom-and-bust’ and ‘man-guided evolution of the rust fungi’ are two of the more memorable phrases that have come to epitomize the failure of race-specific major R genes singly to control fungal diseases of a wide range of major crops including stem and stripe/yellow rust of wheat, late blight of potato, powdery mildew of barley and blackleg in canola. Detailed epidemiological and pathogenicity surveys of such pathogens clearly demonstrate the general tendency for varieties carrying single R genes, to which the local pathogen population had no recent prior experience, to provide protection for only a limited period (e.g. wheat varieties Eureka [*Sr*6], Gabo [*Sr*11], Mengavi [*Sr*36]; Luig and Watson 1970; canola lines containing *LepR*3 resistance; Sprague et al. 2006b). Race surveys reflect this vulnerability with pathogen populations typically responding through one-step or two-step simple genetic changes in infectivity. As a consequence of multiple varietal releases over many years, this can lead to diverse on-going changes in pathogen populations as pathotypes form sequential descendant lineages (Zwer et al. 1992; Steele et al. 2001). In pathogens, whose genetic structure is dominated by continuing cycles of asexual reproduction (e.g. stem rust in Australia and the US Great Plains), selection occurs at the whole-genome level, and lineal descendant pathotypes tend to retain particular infectivity genes even after they are no longer effective.

Compared to race-specific R genes, APR genes are typically thought to provide durable resistance. However, APR genes have rarely (if ever) been deployed alone (possibly reflecting grower concerns regarding the use of ‘dirty crops’ where plants appear infected, albeit showing some field resistance). For example, the wheat variety ‘Hope’, the first commercial variety released carrying the APR gene *Sr*2, also carried the major R genes *Sr*7b, *Sr*9d and *Sr*17 (Hare and McIntosh 1979). Similarly, *Lr*67 (another APR gene present in some of the wheat varieties bred by Norman Borlaug in the 1940s and 1950s) was used in conjunction with major R genes. This is important when considering the durability of APR genes, because the R genes acted to prevent reproduction of all uninfective pathogen isolates (Knott 1990) thereby shielding the *Sr*2 and *Lr*67 genes from direct interaction with the pathogen. As a consequence, while *Sr*2 and *Lr*67 have retained efficacy since their introduction into hexaploid wheat (McIntosh et al. 1995), the independent contribution of these genes to the intensity of selection pressure exerted on pathogen populations cannot be determined. Thus, no formal assessment has been conducted as to whether or not the aggressiveness of associated pathogen races has increased as a consequence of their introduction.

This is not to say that there is no potential for APR genes to provide long-term durability. Both major gene and APR are controlled by the action of single genes, but their molecular nature and role in the biochemical functioning of the plant are very different. Such functional differences may have important consequences for pathogen evolutionary potential (Box 2). Answers to the sorts of questions raised in Box 2 will provide great insights into the potential consequences of pyramiding multiple APR genes into one host line. From a long-term disease control point-of-view, this is of considerable significance given the upsurge in interest in the use of APR genes following the increasing availability of tight-linked or ‘perfect’ molecular markers. If APR genes are directly exposed to pathogen interaction through deployment without R genes, the resultant potentially greater area of cultivation, and hence plant population size, will lead to a substantially increased pressure for future pathogen population changes.

**Box 2: Plant resistance mechanisms and functional constraints on pathogen evolution**

Deploying host resistance mechanisms that are functionally difficult to overcome promises one potential avenue towards durable resistance [see Boyd et al. (2013) and Dangl et al. (2013) for recent reviews describing disease resistance mechanisms in detail and their use in modern agriculture]. Such genetic constraints have long been recognized as generally limiting factors in adaptive evolution (Maynard Smith et al. 1985), and although not typically articulated as such, attempts to leverage developmental constraints underlies much of the thinking regarding potentially durable sources of resistance for agriculture.

**Stacking multiple resistance genes**

This has been widely touted as a means of increasing the degree of functional constraint facing pathogens (Halpin 2005). In particular, resistance gene stacks seek to overcome limitations of single major gene resistance by challenging pathogen populations to accumulate mutations in multiple effector genes (Dangl et al. 2013). Because pathogen populations will not encounter strong selection for new infectivity until all relevant R genes have been overcome, the probability that pathogens will accumulate all relevant mutations (possibly in the face of fitness costs) will be greatly reduced when stacks are deployed. However stacks do not present a mechanistic or functional barrier *per se*, and in the absence of genetic constraints or trade-offs, their durability is likely still dependent on the evolutionary potential of the pathogen in question (McDonald and Linde 2002a).

**Pattern recognition receptors**

One approach to raise the level of functional constraint facing the pathogen is to mine or engineer resistance genes that recognize molecules essential to the pathogen that cannot be deleted or modified without major consequence. For example, all pathogens emit a conserved set of signals that play an essential housekeeping role in pathogen growth and survival [known as pathogen-associated molecular patterns (PAMPS: see glossary, Appendix 1)], such as bacterial flagellin (Felix et al. 1999) or fungal chitin (Wan et al. 2008). Hosts have evolved resistance genes known as pattern recognition receptors (PRRs) that detect PAMPS and initiate an immune response. Hence, most hosts are resistant to most potential pathogen species. However, successful pathogens are able to suppress PRR-based resistance using host-specific effector genes or other virulence factors that suppress PAMP perception, signalling and defence responses in the host plant. Transferral of PRRs into crop plants that lack an orthologous receptor could provide new and durable sources of resistance to established pathogen species. While such targeted engineering approaches are still in their infancy, work in model systems demonstrates the potential utility of this approach. For example, transferring the Arabidopsis pattern recognition receptor gene PRR EF-Tu (EFR) into either *Nicotiana benthamiana* or *Solanum lycospersicum* (tomato) confers previously susceptible plants resistance to a wide range of bacterial pathogens. This gene recognizes the bacterial translation elongation factor EF-Tu, an essential factor for protein synthesis that cannot be simply deleted or modified. However, whether such engineering efforts will confer durable resistance remains to be seen and will likely depend on the complexity of the pathways required to circumvent the PRR, and the ability of pathogens to acquire new infectivity factors via horizontal gene transfer.

**Adult plant resistance genes**

Partial resistance genes found in cereals may prove to be a source of resistance that pathogens find functionally difficult to overcome. For example, in wheat, both major (R) gene and adult plant resistance (APR) are controlled by the action of single genes, but their molecular nature and role in the biochemical functioning of the plant are very different. Unlike R genes that are an intimate part of an effector-receptor interaction process, APR seemingly genes mediate resistance through modifying sugar regulation or signalling (e.g. *Lr 34, Lr46, Lr67*). Furthermore, APR resistance is partial and race nonspecific (often across multiple pathogen species). In the case of *Lr34*, resistance is conferred via three amino acid mutations at the ABC transporter gene (Krattinger et al. 2009). The role of this ABC transporter in plants and/or the pathogen is critical to an understanding of the likely long-term durability of the resistance the mutant gene provides. Does the resistance allele simply disrupt the substrate specificity of the transporter and thereby indirectly starve the pathogen? Or in a susceptible interaction (i.e. with the *Lr34* susceptibility allele) does the pathogen directly ‘highjack’ the cell's sugar transporting mechanism for its own purposes?

**Morphological traits**

Other traits that confer partial or quantitative resistance may also promise to prove difficult to evolve counter adaptations against. In particular, canopy or crop architecture traits that reduce the effective density of foliar tissue or alter the microclimate unfavourable for the pathogen have potential to slow or inhibit pathogen transmission and growth (Ando et al. 2007; Andrivon et al. 2013), possibly in ways that may be difficult to evolve counter adaptations against. For example, wheat lines with genetically determined erect or semi-erect leaves show lower infection by *Bipolaris sorokiniana* (*spot blotch*) than do lines having drooping and semi-drooping leaves, possibly because erect leaves retain less free water, thus making germination of pathogen spores less likely (Joshi and Chand 2002). While the impact of these morphological differences on disease occurrence might be affected by environment (e.g. crop lodging), it is difficult to imagine how pathogen isolates could evolve to greater aggressiveness in response to this character *per se*. Extensive use of these sorts of traits to provide protection against pathogens will need thorough assessment of G × E interactions as their impact may vary across environments and years (Arraiano et al. 2009).

When previously unchallenged R genes are deployed singly into an environment where a significantly large pathogen population is sustained by the presence of susceptible varieties (either varieties with no resistance or those containing resistances that have previously been overcome), and or alternative or alternate hosts, the long-term protection provided will be determined by the product of the effective population size on susceptible varieties, the mutation rate at relevant pathogen infectivity loci and the probability that a novel pathogen mutant survives and increases in the population. The survival of a novel mutant will be determined by competitive fitness interactions and the real possibility of rapid extinction through genetic drift (Barrett et al. 2008). On the other hand, because of the strong directional selection that a highly resistant variety places on the extant pathogen population (leading to the immediate death of uninfective propagules), a novel mutant capable of infecting that variety is at a major selective advantage having a new untapped resource to exploit. Whether or not such novel mutants retain the ability to infect the originating variety is likely to be significantly affected by the pathogen's reproductive mode which in turn determines whether individual genes or the genome as a whole is the target of selection (Burdon and Silk 1997).

### Pyramided genic resistances

The presence of multiple R genes in wheat varieties released in the last few decades would appear to belie the argument regarding difficulties in pyramiding genes based on phenotypic selection (see above). However, in reality, such multigene combinations often arose through sequential insertion of additional R genes into advanced germplasm that, as a consequence of on-going pathogen evolution, was effectively unprotected (only ineffective R genes present) (Jørgensen 1993). Indeed, many wheat lineages in Australia (e.g. ‘Spear’ types) are re-released backcross derivatives of well-adapted varieties containing new rust resistance genes. This situation has now changed, and the availability of very tight chromosomally linked molecular markers (e.g. Mago et al. 2011a; Herrera-Foessel et al. 2012) makes extensive pyramiding a practical reality providing that breeding programmes are supported with basic germplasm enhancement activities aimed at identifying and developing such markers for effective R genes.

Theoretically, three or four R gene stacks should present pathogen populations with major evolutionary hurdles. However, the extent to which that occurs will be dependent on, among other factors, the effective size of the challenging pathogen population and the mutation frequencies of the infectivity genes involved (e.g. Flor 1958). In the immediate term, these R gene stacks will be composed of genes naturally occurring in the crop or its wild relatives. Many of these may have already been used in one or more of the numerous breeding programmes found around the world. As a consequence, it is highly likely that individually each R gene will already have been exposed to a pathogen population somewhere and infectivity may exist in the field, albeit at very low frequencies and some distance from the intended site of deployment.

Combining a number of R and APR genes within the same individual is seen as a means of ensuring some durability of resistance. In such combinations, uninfective pathogen races are not directly exposed to the selective impact of the APR gene due to the overriding pleiotropic effect of the associated R gene(s). The R gene is still subject to challenge by the pathogen, but the presence of the APR gene prevents a full loss of resistance when the R gene is overcome. There is thus a shift in the type of selection pressure being experienced by the pathogen as the R gene is overcome (mortality to fecundity; III.→IV., Fig. 1). However, even though gene pyramids of this type have been used to combat cereal rusts for some years [e.g. *Sr2* with *Sr 7b* & *Sr17* in the Australian variety Warigo released in 1941; or with *Sr6, Sr7b, Sr9d* & *Sr17* in the Canadian variety Selkirk released in 1956 (McIntosh et al. 1995)] and some information can be gleaned about the vulnerability of the R genes (time of exposure etc.), we have no direct knowledge regarding the long-term effectiveness of the APR gene in such evolutionary interactions. Molecular markers with tight linkage to R and APR genes are greatly expanding disease resistance combination options by making complex resistance pyramids readily feasible. As noted above, multiple R gene combinations have been seen at least in some form to date (although because of previous exposure many may be effectively single or two-gene combinations only); other combinations though [such as the pyramiding of multiple APR genes, or gene ‘cassettes’ or stacks involving several novel R genes packaged as a single inheritable unit (Zhu et al. 2012)] have yet to be developed and deployed.

Partial resistance generated by the additive effects of multiple minor resistance genes imposes different selective pressures on the pathogen than does R gene resistance because it limits rather than prevents pathogen growth and reproduction. Furthermore, because it does not display the spectacular failures seen with major R gene deployment, minor gene resistance is often regarded as ‘durable’ and consequently has been used in a wide range of crops (Parlevliet 2002). However, detailed studies of such quantitative systems have demonstrated that partial resistance often does not prevent pathogen evolution. Rather, there is growing evidence for increased aggressiveness and adaptation by pathogen isolates to partially resistant host lines and thus sequential reductions in the disease control benefit gained from such resistance. For example, comparisons of the aggressiveness of different isolates of *Mycosphaerella graminicola* collected early and late in epidemics on susceptible and partially resistant (minor resistance genes only) wheat cultivars demonstrated that partial resistance selected for more aggressive isolates leading to greater leaf areas infected than did susceptible host lines (Cowger and Mundt 2002). Results from assessments of aggressiveness in other quantitatively based systems involving *Phytophthora infestans* (Andrivon et al. 2007), lettuce mosaic virus (Pink et al. 1992), potato virus Y (Montarry et al. 2012) and nematodes (Schouten and Beniers 1997) found similar results with these pathogens all showing increasing levels of aggressiveness on partially resistant host lines.

Quantitative resistance to pathogens may be derived from a very wide array of physical and biochemical features (Poland et al. 2008). As a consequence, there are likely to be differences between partial resistance sources in the extent of G × E effects and in the extent of evolutionary interaction between specific quantitative resistance mechanisms and pathogen aggressiveness. Subsequent interactions with host genetic background and resulting genic expression may be large (see Box 2).

### Physical mixtures

There is an extensive literature on the impact of varietal mixtures and multilines on crop performance (Finckh et al. 2000). Mixtures of two or more varieties carrying different, usually major R gene resistance profiles have repeatedly been shown to reduce the incidence and severity of a wide range of biotrophic and necrotrophic diseases attacking many different crop hosts (including wheat, barley, rice, potatoes, sugar beet, canola, apples and coffee: see review by Finckh and Wolfe 2006). However, the extent of disease reduction is often environmentally dependent (Mundt 2002) being greatest under conditions of low-to-moderate inoculum pressure (Pilet et al. 2006) and high auto-infection, while being limited or not apparent under conditions conducive to pathogen development (Klein and Marshall 1989) when inoculum production and dispersal are sufficient to generate significant epidemics.

Although the major focus of studies of pathogen–host interactions in mixtures has been on disease development, detailed empirical studies of the genetic structure of pathogen populations have detected changes in both infectivity and aggressiveness within crop mixtures compared to that observed in relevant pure stands. Thus, pioneering work on barley mildew populations demonstrated that under the selective regime imposed by a three-component mixed host population, the range of infectivity possessed by pathogen genotypes depended on the relative fitness of each pathotype across all host lines (Chin and Wolfe 1984). In some pathogen–host combinations, unnecessary infectivity was rapidly selected against but less so in others. In essence, selection for pathotypes with broad infectivity ranges limited increased aggressiveness on individual host lines. This, combined with reductions in the size of the total pathogen population in mixtures, reduced the absolute frequency of occurrence of pathotypes with multiple infectivity genes in mixtures relative to that in the component pure stands. Experiments involving mixtures of stripe rust on wheat cultivar mixtures found a similar reduction in the absolute frequency of complex races in mixtures as compared with pure stands (Dileone and Mundt 1994). Finally, Kolmer (1995) constructed three- to six-line multiline varieties each composed of various proportions of the wheat variety Thatcher and near-isogenic lines differing in the major leaf rust resistance genes they carried. These mixtures were challenged with a heterogeneous population of *Puccinia recondita* f.sp. *tritici* derived from a sexual recombination event. Over 12 uredinial generations of selection, races with an intermediate infectivity profile appeared to have fitness advantage relative to other isolates (either more or less infective) on the three host multilines.

Overall, the effect of host mixtures on pathogen infectivity and aggressiveness is likely to be quite complex and unpredictable. Some of these effects will be driven by a range of genetic and environmental factors (e.g. dispersal patterns and the relative frequency of auto- to allo-infection), while others will be dependent on the population context (e.g. the fitness of competing pathogen races) or be isolate specific (e.g. the extent of fitness costs associated with specific infectivity factors). Mundt (2002) succinctly states ‘After several decades of study we still do not know the rate at which [selection towards increased relative frequency of complex races in host mixtures] will occur or to what degree, if any, it would decrease the disease control provided by mixtures’.

The evolutionary response of pathogens to selection by mixtures and multilines involving limited host resistance has been extensively modelled for situations involving R genes. Depending on the level of ecological realism involved (spatially explicit dispersal; fitness costs associated with infectivity etc.), such models generally predict pathogen populations in mixtures to be smaller with a pathogenically more diverse (more races) but simpler (lower infectivity pattern) population structure than those occurring in relevant pure stands (Barrett 1980; Lannou and Mundt 1997; Lannou 2001), while levels of aggressiveness also vary (Lannou 2001; Marshall et al. 2009). Using a population genetics model to assess interactions between aspects of the genetic and spatial composition of host populations and their effect on pathogen populations, Sapoukhina and colleagues (Sapoukhina et al. 2009) demonstrated that random patterning of monogenic resistances in a mixture could match the epidemic control generated through the use of pyramided multigenic resistance. Patchy distributions of hosts on the other hand lead to pathogen diversification and reduced efficacy of the R genes. Similarly, using a spatially explicit gene-for-gene model involving major resistance alleles conferring either complete or partial resistance to specific pathotypes, Nemri and colleagues (A. Nemri, J. J. Burdon, M. E. Hochberg and P. H. Thrall, unpublished manuscript) found that these two classes of resistance could act synergistically when deployed among host individuals, leading to reduced disease epidemics and a significant slowing of pathogen evolution towards increased infectivity.

These and earlier global dispersal models demonstrated that the way in which R genes were deployed in mixtures (individually, as overlapping or as disjoint sets of genes) could affect the evolution of complex races (Marshall and Pryor 1985; Marshall and Weir 1985). Despite this, little attempt has been made to extend this line of approach to assess the diversity of combinations of major R genes, combinations of R genes and minor gene resistance, or R genes and APR genes now becoming available with regard to their potential to control disease development and pathogen evolution.

## On-farm and regional spatial and temporal deployment strategies

The spatial scale of resistance gene deployment strategies runs from regional allocation of resistance genes through on-farm variation generated by few large fields versus multiple small fields, to intercropping within individual fields and the use of random mixtures in an individual crop (Papaïx et al. 2011). At the same time, deployment strategies may vary from continuous cropping of the same variety to a wide range of temporal rotation practices that inject different species with quite different pathogens into a regulated sequence. In general, not all strategies work for all pathogen–crop combinations nor is the efficacy of any one strategy constant through time or space. Indeed, the spatial scale at which gene deployment strategies reduce epidemic development is determined by host and pathogen traits interacting with the environment. For the pathogen, this includes dispersal mode, presence of alternative hosts and off-season survival, while for the host, it includes the genetic structure of the population, ontogenetic development and plant density (Papaïx et al. 2014).

A significant number of studies have assessed aspects of on-farm spatial patterning of crops (intercropping, interfield diversification etc.) and have demonstrated reductions in epidemic development (e.g. late blight in potato, Bouws and Finckh 2008; *Ascochyta* blight in peas, Schoeny et al. 2010; see Boudreau 2013 for review of intercropping). In addition to the immediate value of such strategies in reducing the impact of disease in a crop through reductions in dispersal and within-season temporal delays in epidemic peaks, reductions in the size of pathogen populations may potentially lead to a slowing of evolutionary potential. However, while this theoretically could occur through a reduction in the absolute number of mutation events or through losses in diversity (via drift), as yet no empirical studies have attempted to assess this longer-term evolutionary impact.

Farm management also plays a critical role in determining resistance durability. Thus, resistance to some pathogens may be durable because the farming system prevents the build up of inoculum thereby reducing the chances of adaptation in the pathogen population (Parlevliet 1993). For biotrophic pathogens, the loss of green bridges maintained by volunteers or other weedy hosts may lead to severe local bottlenecks or extinction of pathogen populations with concomitant loss of locally adapted (infective) races. For necrotrophic fungi, crop rotation may prolong the period during which pathogen populations are responding to distinctly different selective pressures. For example, for foliar diseases such as barley scald, this may result in an extended period of saprophytic growth or, in the case of soilborne pathogens, may result in marked changes in the physico-chemical environment as a result of changed root exudates. Even where rotations are simply restricted to different varieties of the same species (e.g. carrying different R genes), this can, in some circumstances, result in lower levels of disease, increased longevity of specific R genes and significant changes in the structure of the resident pathogen population (Marcroft et al. 2012).

Finally, regional gene deployment strategies have been proposed as a means of controlling pathogens that disperse long distances over well-defined pathways and generally whose full geographic distribution has to be re-established annually. While such an approach has been advocated for cereal rusts in the United States and for barley powdery mildew and potato late blight in Europe (Finckh and Wolfe 2006), and a few models have been developed (e.g. Parnell et al. 2006), the potential for developing new host resistance management strategies at the landscape scale remains largely unexplored (Plantegenest et al. 2007). However, by analysing large datasets describing the interaction between wheat and wheat leaf rust across the whole of France, Papaïx and colleagues (Papaïx et al. 2011) established a link between field resistance levels of varieties and varietal composition across the landscape. This suggested the potential for significant impacts on the infectivity structure of pathogen populations in different areas. Furthermore, pathogen adaptation to broad environmental variables (particularly temperature) is one factor that could be exploited to support the use of regional gene deployment strategies. Thus, comparisons between wheat-infecting *P. striiformis* populations occurring in northern and southern France show the existence of clear differences between the two pathogen populations in quantitative traits (Mboup et al. 2012).

In reality, while on-farm and regional deployment strategies hold theoretical appeal and some practical benefits, such approaches need also to be evaluated from the perspective of what is feasible for farmers to implement (McDonald 2010) as well as their impacts on profitability. For regional deployment strategies, the controls that would be needed around farmer-to-farmer seed trading and the use of farmer-saved seed, and agreements from breeders to forgo using some resistance resources are very substantial. While on-farm strategies are generally likely to be simpler to implement, regardless of their effectiveness in reducing disease and constraining evolution, such strategies must still be agronomically, technologically and economically feasible. However, at the same time, it is important to recognize the speed of practice change on farms – changes that will continue apace as new geo-spatial technologies drive next generation precision agriculture.

## Conclusions and future issues

Too often, agriculture has shown boundless enthusiasm for new technologies without giving due consideration to the potential for unintended consequences arising as a result of evolutionary responses from targeted organisms (Thrall et al. 2011). Thus, the appearance of fungicide resistance in crop pathogens and of herbicide resistance in many agricultural weeds was an evolutionary inevitability given the unsophisticated way fungicides and herbicides have often been used. However, such failings are not inevitable. For example, application of sound ecological and evolutionary principles has maintained the efficacy of *Bt*-based resistance in Australian cotton to cotton bollworm *Helicoverpa punctigera* (Downes and Mahon 2012).

The on-going battle to control the effects of plant pathogens through the deployment of resistance has a long history of success and disappointment as resistances have been used with relatively little thought as to the combined epidemiological and evolutionary consequences of particular strategies. Without doubt, greater knowledge of fitness costs associated with mutations affecting pathogen infectivity or aggressiveness is critical to predicting the evolutionary trajectories of plant pathogens (Leach et al. 2001; Lannou 2012; Zhan and McDonald 2013). In this regard, detailed analyses of:

1. the extent of variation in aggressiveness in a set of pathogen–host combinations representative of economically important biotrophic and necrotrophic pathogens;
2. the rate of temporal change in aggressiveness resulting from continued cycling of specific pathogen isolates on specific host lines; and
3. the extent to which adaptation in aggressiveness is disrupted by periodic growth on different host lines

Are essential to developing a clear understanding of the level of dynamism in traits that affect aggressiveness. This information lies at the core of any assessment of the long-term ability of partial resistance to slow evolutionary change in the pathogen.

**Figure 3:**
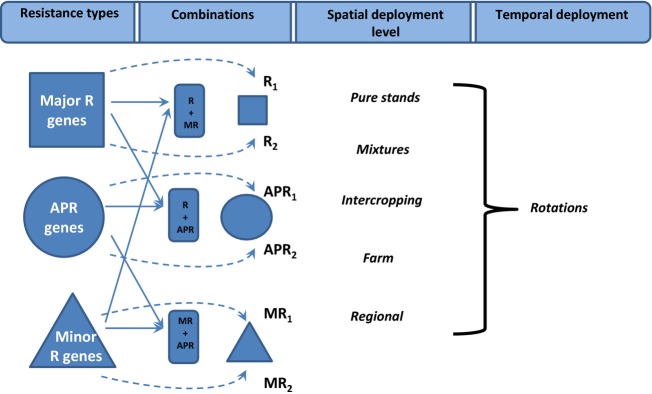
Summary of the different types of resistance, ways in which they may be combined within host lines, and the ways in which the consequent germplasm may be deployed both spatially and temporally.

Complementing this approach is the current drive to understand the molecular control of both resistance and infectivity and how this may assist in devising new durable resistance strategies (Dangl et al. 2013; Michelmore et al. 2013; Hulbert and Pumphrey 2014). Rapid advances in molecular marker technologies are revolutionizing the ways in which resistance can be manipulated in breeding programmes. As a consequence, it is essential to rigorously apply evolutionary principles to deployment strategies – of different combinations of R genes, of R genes and minor gene resistance or of R genes and APR genes in different spatial and temporal settings (Fig. 3). Each of these forms of resistance place different selective pressures on their associated pathogen populations and thus may drive the evolution of pathogen infectivity and aggressiveness in different directions and at different rates. In this respect, APR genes are perhaps least well understood despite the fact that they are widely regarded as important contributors to resistance durability in many wheat cultivars. Elucidating the mode of action of APR genes should be a high priority as they are likely to become an increasingly important component of long-term disease control strategies and efforts are made to mimic their effect in species in which they are currently unknown.

There is increasing evidence that careful use of all of these different types of resistance can generate deployment strategies with the potential to simultaneously reduce short-term epidemic development and the probability of longer-term evolutionary change in the pathogen. Successful execution of this promise requires the development of agriculturally realistic simulation models based on evolutionary principles and guided by detailed knowledge of pathogen biology and life history. Predictions from such modelling approaches then need to be the focus of realistic experimental assessments.

Finally, it is essential to ensure that resistance strategies are integrated into whole crop and farm management systems. In reality, despite their potential, it is likely that not all biologically beneficial deployment strategies will be economically or politically feasible. However, rapid advances in remote spatial control (e.g. remote sensing, global positioning), of crop–climate models and of planting and harvesting technologies raise the possibility of future farming approaches that are far more amenable to dealing with close intercropping or even mixtures (separation technologies) than is currently possible. In summary, we argue that development of a robust predictive framework for evaluating the epidemiological and evolutionary consequences of different genetic and spatio-temporal deployment options in the context of what is feasible from a farming perspective represents a major research goal with real application potential for managing disease.

## Appendix 1

### Glossary

Adult plant resistance (APR): partial resistance detected at postseedling stages of development in cereals, which is controlled by single genes effective against all races or pathotypes of a pathogen (i.e. race nonspecific). Often such genes are effective against more than one pathogen species.

Aggressiveness: a quantitative measure of pathogen growth and development in the host. Multiple traits including infection efficiency, toxin production, latent period, infectious period and spore production contribute to differences in the aggressiveness of different pathogen isolates.

Biotroph: a pathogen that is entirely dependent on its host for growth and nutrients. Biotrophs keep their hosts alive during part or all of the infection cycle.

Boom-and-bust: Cycles of rapid increasing deployment of specific crop varieties (protected by specific major resistance genes) followed by their ‘collapse’ as evolution in the pathogen population for matching infectivity leads to an effective loss of resistance and an upsurge in disease epidemics.

Durable resistance: Resistance that persists. Durability is a trait that currently can only be measured in retrospect (i.e. after it is no longer effective). It is often assumed to be an inherent feature of nonrace-specific resistance although this is not always the case.

Infectivity: whether or not a pathogen isolate can infect a given host line and generate a fully compatible reaction. Infectivity has the same meaning as ‘virulence’ as defined by van der Plank (1963) and widely used in the plant pathology literature. However, the use of ‘virulence’ in this context is easily confused with its use in animal and human pathogen circles and its usage in everyday speech where it means ‘aggressiveness’.

Major gene resistance (R): race- or pathotype-specific resistance controlled by genes with large phenotypic effects. Such genes are typically (but not exclusively) associated with Flor's gene-for-gene system.

Minor gene resistance: partial resistance controlled by many individual genes each of which have small phenotypic effects.

Necrotroph: a pathogen that kills host tissue as it colonizes it.

PAMP: Pathogen-associated molecular patterns; phylogenetically and functionally conserved molecular motifs, such as chitin and flagellin that are recognized by plants as signals of pathogen attack.

Pyramid (gene): the accumulation of two or more resistance genes in the one host line with the intention of increasing the time that elapses before a pathogen may be able to generate a fully susceptible infection reaction.

Race-specific resistance: host resistance that is expressed against some, but not other, races or pathotypes of a pathogen.

Race-nonspecific resistance: host resistance that is expressed against all races or pathotypes of a pathogen.

Virulence: see ‘infectivity’.
